# The need for future research into the assessment and monitoring of eating disorder risk in the context of obesity treatment

**DOI:** 10.1002/eat.23898

**Published:** 2023-01-24

**Authors:** Caitlin M. McMaster, Susan J. Paxton, Sarah Maguire, Andrew J. Hill, Caroline Braet, Anna L. Seidler, Dasha Nicholls, Sarah P. Garnett, Amy L. Ahern, Denise E. Wilfley, Natalie B. Lister, Hiba Jebeile

**Affiliations:** ^1^ The University of Sydney Children's Hospital Westmead Clinical School Westmead New South Wales Australia; ^2^ School of Psychology and Public Health La Trobe University Melbourne Victoria Australia; ^3^ InsideOut Institute for Eating Disorders, Boden Collaboration for Obesity, Nutrition and Eating Disorders Charles Perkins Centre, The University of Sydney Sydney New South Wales Australia; ^4^ Leeds Institute of Health Sciences University of Leeds Leeds UK; ^5^ Department of Developmental, Personality and Social Psychology Ghent University Ghent Belgium; ^6^ National Health and Medical Research Council Clinical Trials Centre The University of Sydney Sydney New South Wales Australia; ^7^ Division of Psychiatry Imperial College London London UK; ^8^ Kids Research Sydney Children's Hospital Network Westmead New South Wales Australia; ^9^ MRC Epidemiology Unit University of Cambridge Cambridge UK; ^10^ Washington University in St. Louis St. Louis Missouri USA

**Keywords:** assessment, feeding and eating disorders, obesity, overweight, risk factors, weight management

## Abstract

In adolescents and adults, the co‐occurrence of eating disorders and overweight or obesity is continuing to increase, and the prevalence of eating disorders is higher in people with higher weight compared to those with lower weight. People with an eating disorder with higher weight are more likely to present for weight loss than for eating disorder treatment. However, there are no clinical practice guidelines on how to screen, assess, and monitor eating disorder risk in the context of obesity treatment. In this article, we first summarize current challenges and knowledge gaps related to the identification and assessment of eating disorder risk and symptoms in people with higher weight seeking obesity treatment. Specifically, we discuss considerations relating to the validation of current self‐report measures, dietary restraint, body dissatisfaction, binge eating, and how change in eating disorder risk can be measured in this setting. Second, we propose avenues for further research to guide the development and implementation of clinical and research protocols for the identification and assessment of eating disorders in people with higher weight in the context of obesity treatment.

**Public Significance:**

The number of people with both eating disorders and higher weight is increasing. Currently, there is little guidance for clinicians and researchers about how to identify and monitor risk of eating disorders in people with higher weight. We present limitations of current research and suggest future avenues for research to enhance care for people living with higher weight with eating disorders.

## 1 INTRODUCTION

People with obesity have elevated levels of disordered eating behaviors (e.g., binge eating, self‐induced vomiting, laxative misuse) (Hayes et al., 2018; He et al., 2017; Nightingale & Cassin, 2019) and have a higher prevalence of eating disorders (EDs) compared to lower weight peers, although this varies by sample (Duncan et al., 2017; Flament et al., 2015; Mitchison et al., 2019; Udo & Grilo, 2018). A systematic review of behavioral weight management, pharmacotherapy, and bariatric surgery in children and adolescents found between 6% and 71% reported prevalence of binge eating or loss of control at baseline (Moustafa et al., 2021). Indeed, co‐occurrence of obesity and recurrent binge eating increased 5.7‐fold between 1995 and 2015 in a community sample of Australian adults (Da Luz et al., 2017).

Whether ED scores and symptoms resolve, are maintained, or increase following weight management has been studied. Recent reviews found evidence‐based, supervised obesity interventions do not increase ED risk up to 2‐years from baseline, with fewer studies reporting data at 3‐, 4‐, and 6‐years (Da Luz et al., 2015; Jebeile et al., 2019; Moustafa et al., 2021; Peckmezian & Hay, 2017). Thus, concerns over ED risk should not prevent access to obesity treatment. However, there may be a subset of people presenting for obesity treatment with an undiagnosed ED, who may experience the onset of new disordered eating attitudes or behaviors, or may develop an ED during or following obesity treatment. There is insufficient data to compare the rate of onset of EDs or disordered eating attitudes or behaviors in obesity treatments settings compared to the community, and further investigation is warranted.

While clinical practice guidelines for management of EDs recognize that people with higher weight are at risk (Hay et al., 2014; Ralph et al., 2022), there is little guidance on how to assess and monitor ED risk in obesity treatment settings. This is particularly important, as data from a systematic review of 14 studies suggest that people with an ED are more likely to seek weight loss treatment (30%–73%) than ED treatment (23%) (Hart et al., 2011). In the context of obesity treatment, ED assessment aims to identify people with a clinical ED requiring referral to a treatment service, those who may experience the onset of new symptoms during obesity treatment or with disordered eating attitudes and behaviors that may worsen during obesity treatment rather than improve. Therefore, in this context, ED risk is considered to be the likelihood that symptoms of an ED, scores on assessments or frequency of disordered eating behaviors, or associated distress, may worsen as an adverse treatment outcome. For people seeking treatment for obesity, this potential risk needs to be balanced with potential benefits of obesity treatment at the individual level. The current literature regarding the assessment of ED risk in obesity treatment setting has several limitations. Therefore, this article aims to: (1) summarize current challenges and knowledge gaps regarding assessment of ED risk among people with higher weight seeking obesity treatment; and (2) propose research to address these gaps and enhance care for people with higher weight seeking obesity treatment at risk of EDs.

Within this Spotlight, obesity treatment settings are defined as any service providing a treatment plan or program that includes weight loss or weight maintenance as one possible outcome. This may include behavioral weight management, pharmacotherapy, and/or bariatric surgery to address high weight alone or as part of the treatment plan for another chronic disease where health improvement may be achieved with weight loss, e.g., type 2 diabetes. Settings may include commercial programs, primary care, or specialist multidisciplinary weight management clinics. Service provision varies greatly by jurisdiction and across differing health systems. Thus, within this manuscript we discuss general principles relating to the assessment, monitoring, and measurement of ED risk in people with higher weight seeking obesity treatment. Further research is needed to understand how assessment processes can be adapted to different settings and forms of treatment.

The identification of EDs in people with higher weight who are not seeking weight management, or in settings where weight may be discussed without a patient seeking this, are also important. However, discussion of these are beyond the scope of this manuscript.

## 2 CURRENT CHALLENGES

Approaches to assess and monitor for EDs in adolescents (Jebeile et al., 2021) and adults (D'Adamo et al., 2023; Schutz et al., 2019) with higher weight in obesity treatment settings have been published but not evaluated. These including screening for EDs as part of history taking and prior to obesity treatment, using open‐ended questions relating to overvaluation of shape and weight, binge eating and unhealthy weight control behaviors and monitoring for the emergence of risk factors such as cognitive rigidity (Braet et al., 2014; Goldschmidt et al., 2008; Lister et al., 2020; Rancourt & McCullough, 2015; Schutz et al., 2019; Taylor et al., 2018). A staged approach using self‐report questionnaires, followed by a clinical assessment or interview has also been suggested (Braet et al., 2014; D'Adamo et al., 2023; Lister et al., 2020). The 2022 US Preventative Services Task Force Recommendation Statement on Screening for Eating Disorders noted the need for further research into the potential benefits and harms of early detection and intervention (US Preventive Services Task Force, 2022). Of relevance to obesity treatment is the potential impact of false‐positive ED screening results which may prevent referral to obesity services. As ED services for people with higher weight are limited in many jurisdictions, this may leave patients without access to any care, adding undue distress.

Numerous ED self‐report assessments are available (Hay et al., 2022); however, those assessing global ED risk have been predominantly developed without consideration of the unique needs of people with higher weight and have primarily been validated in people with body mass index (BMI) <25 kg/m^2^. In obesity treatment settings, resources are scarcely available to conduct diagnostic interviews to comprehensively assess ED risk. Hence, self‐report assessments to enable screening and monitoring of ED risk are preferred. There are several considerations regarding the appropriateness for use with people with higher weight.

### 2.1 Validation of current measures

A 2022 review identified 27 studies (22 adult, 5 adolescent) validating 15 ED self‐report questionnaires against diagnostic interview in samples with overweight or obesity (House et al., 2022). Most studies screened for binge eating or binge eating disorder (BED) with few questionnaires validated to identify the full spectrum of ED diagnoses. The Eating Disorder Examination‐Questionnaire (EDE‐Q; sensitivity = 0.16–0.88, specificity = 0.62–0.89) and Questionnaire on Eating and Weight Patterns (QEWP‐R; sensitivity = 0.07–0.88, specificity = 0.63–0.93) were most frequently validated (six studies each). The Binge Eating Scale (BES) and the QEWP‐R showed better diagnostic accuracy, compared to other self‐report assessments, in identifying BED (House et al., 2022). Importantly, ED assessments may perform differently in people with higher weight. For example, the original subscale structure (dietary restraint, shape concern, weight concern, and eating concern) used with the EDE‐Q and Eating Disorder Examination interview has been found to have an inadequate fit in samples with class 2 or 3 obesity (BMI≥35 kg/m^2^). Instead a 7‐item 3‐factor structure (dietary restraint, shape/weight overvaluation, and body dissatisfaction) has shown better fit (Grilo et al., 2010; Grilo et al., 2013). However, this scoring system is not widely used.

Below we discuss three aspects of self‐report assessments to consider when determining appropriateness for use in populations with higher weight in obesity treatment settings.

### 2.2 Dietary restraint

Dietary restraint is an established risk factor for binge eating and EDs in community samples (Neumark‐Sztainer et al., 2006; Stice et al., 2011), and dietary change with or without energy restriction is often core to weight management. Dietary restraint or dieting cannot be categorized as entirely healthy or unhealthy but could be health‐promoting or detrimental depending on the circumstance, form and available support (Haynos et al., 2015; Schaumberg & Anderson, 2016). For example, during pediatric obesity treatment, measures of dietary restraint increase or remain unchanged, while binge eating and global ED scores do not increase (House et al., 2021). Therefore, current measures of dietary restraint may not be adequate markers of ED risk in this context (Stewart et al., 2022).

In the literature, dietary restraint is often described as flexible (graduated approach, foods are eaten in limited quantities without guilt, rather than being eliminated) or rigid restraint (dichotomous, rule‐driven, all‐or‐nothing approach to eating), with the latter perceived to be more indicative of pathological eating patterns (Westenhoefer et al., 1999). In an obesity treatment context, flexible restraint may include components of behavioral weight management such as reducing snacking between meals, intake of energy‐dense foods, non‐hungry eating or emotional eating. Prescriptive dietary interventions such as low or very low energy diets may be considered a form of rigid restraint. Current evidence suggests that both of these dietary approaches result in small reductions in ED risk, with the onset of binge eating reported in a small subgroup of participants in studies reporting individual variation in response (Da Luz et al., 2015; Jebeile et al., 2019). It is likely that there is individual variation in the experience of both flexible and rigid restraint, and potential development of disordered eating and EDs.

Research is required to understand what degree of dietary restraint may facilitate management of health conditions without worsening ED risk, and specifically: (1) Can we understand which types of restraint are helpful or harmful for different people?; (2) Do current measures of dietary restraint differentiate between dieting behavior and dietary restraint cognitions?; (3) When does dietary restraint move from being helpful to harmful?; and (4) When does it reflect an increase in ED risk?

### 2.3 Body dissatisfaction

Core to ED assessment is the ability to differentiate between individual perception of weight and body dissatisfaction, and overvaluation of weight and shape, i.e., judging one's self‐worth exclusively or predominantly in terms of their weight and shape. While overvaluation of weight and shape is known to be associated with ED psychopathology independent of weight, it is not clear whether body dissatisfaction alone is a predictor of disordered eating in people with higher weight (Loth et al., 2015). Indeed, data show that people with higher weight have higher body dissatisfaction than their lower weight peers (Moradi et al., 2021; Weinberger et al., 2016). Presentations of body dissatisfaction in people with higher weight may differ from people with lower weight and be influenced by factors that are not routinely assessed by current self‐report assessments, e.g., experience of weight stigma, pain or limited mobility. These are often included in assessment of weight‐related quality of life (Kolotkin et al., 2001; Kolotkin & Crosby, 2002). Incorporating items on internalized weight bias, pain, and functional problems into ED assessment may strengthen the interpretation of body dissatisfaction in this population.

### 2.4 Binge eating

Assessment of binge eating is characterized by eating a large amount of food, in relation to peers, with a sense of loss of control. People with higher weight generally have higher energy requirements (consuming larger food volume) compared to people with lower weight. Defining what is a “large amount of food” among people with differing weight status is challenging, particularly using self‐report, and likely to vary by developmental stage and gender (Tanofsky‐Kraff et al., 2020). This may contribute to overreporting of disordered eating when using self‐report assessments compared to clinical interview (Mond et al., 2004). Moreover, studies have suggested that loss of control is a better marker of disordered eating compared to volume of food consumed (Colles et al., 2008; Shomaker et al., 2010) and is associated with weight gain and psychological distress (Tanofsky‐Kraff et al., 2004; Tanofsky‐Kraff et al., 2009).

The BES and QEWP likely provide the most accurate self‐report assessment of BED as they were developed for people with higher weight (House et al., 2022). Assessments such as the EDE‐Q often require a clinical interview to accurately quantify the frequency and size of binge eating episodes, the resources for which are unlikely to be available in obesity services. The addition of instructions to the EDE‐Q helps quantify the number of days of binge eating (Celio et al., 2004). Future self‐report assessments of global ED risk in people with higher weight would benefit from additional instructions defining a large amount of food and loss of control. This would improve assessment of overeating without loss of control, which may be an antecedent of weight gain and predispose someone to engage in extreme dieting or compensatory behaviors.

## 3 CONSIDERATIONS FOR MEASURING CHANGE IN EATING DISORDER RISK

Measuring change in ED risk is needed to understand the safety and efficacy of obesity treatment. This may include assessing change scores in disordered eating attitudes and behaviors as a continuous variable, or a categorical variable using cut‐points to determine if a person has moved above or below a subthreshold or diagnostic level. Table 1 summarizes commonly used ED self‐report assessments and evidence for use in people with higher weight.

**TABLE 1:** Summary of available literature to guide the assessment of eating disorder risk in individuals with higher weight as a categorical variable.

Eating disorder outcome measure	Literature to guide determination of cut point	Considerations
Binge Eating Scale	Cut point of 17 recommended based on sensitivity of ≥0.85 when used to detect BED in adults with obesity (Grupski et al., 2013)	Tool has been validated against diagnostic interview in adult populations with overweight and obesity (Freitas et al., 2006; Grupski et al., 2013; Quilliot et al., 2019; Ricca et al., 2000)
Eating Disorder Examination/Children's Eating Disorder Examination	No literature identified regarding cut‐point in individuals with higher weight Mean scores in samples with obesity: *Sample of adults seeking bariatric surgery* (mean BMI = 52.2) (Kalarchian et al., 2000) Restraint subscale *M* (SD) = 1.60 (1.5) Eating concern subscale *M* (SD) = 1.34 (1.4) Weight concern subscale *M* (SD) = 3.30 (1.1) Shape concern subscale M (SD) = 3.28 (1.4) Global score^a^ *M* = 2.38 *Sample of adults with obesity* (mean BMI = 33.9) (Wilfley et al., 2000) Restraint *M* (SD) = 1.7 (1.3) Eating concern *M* (SD) = 0.6 (0.9) Weight concern *M* (SD) = 1.9 (1.2) Shape concern *M* (SD) = 2.0 (1.3) Global score^a^ *M* = 1.55 *Sample of adolescents seeking inpatient treatment for obesity* (mean BMI as %50th BMI percentile for age and sex = 172%) (Decaluwé & Braet, 2004 **)** Restraint subscale *M* (SD) = 0.96 (0.95) Eating concern subscale M (SD) = 0.63 (0.75) Weight concern subscale *M* (SD) = 1.85 (1.08) Shape concern subscale *M* (SD) = 1.80 (1.27) Global score^a^ *M* = 1.31 Global score comparable to sample of nontreatment‐seeking children and adolescents with overweight who endorsed subjective or objective binge eating (global score *M* [SD] = 1.1 [0.86]) (Tanofsky‐Kraff et al., 2004)	Studies have shown that individuals with overweight or obesity tend to score higher on weight and shape concern subscales compared to restraint and eating concern subscales (Decaluwé & Braet, 2004; Kalarchian et al., 2000; Wilfley et al., 2000) A 7‐item 3‐factor structure appears to have better fit than the original subscale structure (Grilo et al., 2010; Grilo et al., 2013)
Eating Disorder Examination Questionnaire/Child Eating Disorder Examination Questionnaire/Youth Eating Disorder Examination Questionnaire	*Suggested EDE‐Q cut points for adults based on sample of female control participants and patients from specialty eating disorder treatment centers aged 16–66* (Rø et al., 2015): BMI 25–30: 3.15 BMI >30: 3.26 Comparable to cut point of 3.1 in individuals with BMI >25 recommended by Mond et al. (2008) *Sample of adolescents seeking inpatient treatment for obesity* (mean BMI as %50th BMI percentile for age and sex = 172%) (Decaluwé & Braet, 2004): Restraint *M* (SD) = 1.21 (1.00) Eating concern *M* (SD) = 1.39 (1.06) Weight concern *M* (SD) = 2.76 (1.28) Shape concern *M* (SD) = 2.73 (1.51) Global score^a^ *M* = 2.02	Tool has been validated against diagnostic interview in adult and adolescent populations with overweight or obesity (Aardoom et al., 2012; Kalarchian et al., 2000; Mond et al., 2008; Parker et al., 2016) Studies have shown that individuals with overweight or obesity tend to score higher on weight and shape concern subscales compared to restraint and eating concern subscales (Rø et al., 2012) A 7‐item 3‐factor structure appears to have better fit than the original subscale structure (Grilo et al., 2010; Grilo et al., 2013)
Eating Attitudes Test/Children's Eating Attitudes Test	In studies including individuals with overweight or obesity, an optimal cut‐point of 10 or 11 was identified to obtain a balance between sensitivity and specificity (Orbitello et al., 2006; Siervo et al., 2005). This reduced the false negative rate from 68% at the usual cut‐point of 20 down to 32% but gave false positive rate of 35% and overall misclassification rate of 33% (Orbitello et al., 2006). In a community sample of girls aged 9–13 years, using a cut off score of 20, the ChEAT demonstrated a sensitivity of 17%, specificity of 98%, positive predictive value of 63%, and negative predictive value of 87% compared to ChEDE. Decreasing the cut‐off score improved the sensitivity, which reached 76% with a cut‐off of 5; however, the specificity then fell to 59% (Colton et al., 2007). Erickson and Gerstle (2007) found the sensitivity of the ChEAT was overall low and varied with age based on use in adolescent sample aged 8–12. Using a threshold of 25, overall sensitivity was 36%; however, this increased to 53% among girls aged between 10 and 12 years.	Validated against diagnostic interview in adult population overweight or obesity (1 study with sample containing individuals in normal weight range and higher BMI) (Orbitello et al., 2006)
Eating Disorder Inventory	No literature identified regarding cut‐point in individuals with higher weight. In a community sample of women aged 22–27 years (weight status not reported), best screen for BED was the global score of three subscales (Bulimia, Drive for Thinness, Body Dissatisfaction) with an AUC of 0.86 (Mustelin et al., 2016). Sensitivity was 87% and specificity 76% at cut‐off ≥21. Three individual subscales had acceptable screening properties: Bulimia (AUC 0.83; sensitivity 80%, specificity 78% at cutoff ≥2) Drive for thinness (AUC 0.82; sensitivity 87%, specificity 72% at cutoff ≥7) Body dissatisfaction (AUC 0.81; sensitivity 93%, specificity 60% at cutoff ≥8)	Not validated against diagnostic interview in population with higher weight
Questionnaire for Eating and Weight Patterns/Questionnaire for Eating and Weight Patterns‐Adolescent Version	In adult sample participating in BED treatment study with mean BMI 33.6, cut point of 2 demonstrated reasonable sensitivity in identifying individuals with BED (0.74) (Celio et al., 2004). Authors suggested QEWP is adequate for screening purposes, however, sole reliance for diagnosis of BED is not recommended due to high percentage of misclassifications. Not recommended for determining the size of an eating episode or ability to define loss of control eating in children with or without an ED (Tanofsky‐Kraff et al., 2003).	Validated against diagnostic interview in adults with overweight or obesity (Borges et al., 2005; Calugi et al., 2020; De Zwaan et al., 1993; Dymek‐Valentine et al., 2004; Parker et al., 2016)
Sick Control One Fat Food (SCOFF)	In sample of adults attending a psychiatric outpatient clinic with a BMI ≥27, cut point of 2 recommended for males (AUC 0.66) and 3 for females (AUC 0.90) (Liu et al., 2015). Using usual cut‐point of 2 has demonstrated sensitivity of between 0.67–1.0 and specificity of 0.59–0.7 in individuals with overweight or obesity (Liu et al., 2015; Mond et al., 2008; Solmi et al., 2015)	Validated against diagnostic interview in adults with overweight or obesity (Liu et al., 2015; Mond et al., 2008; Solmi et al., 2015).

Abbreviations: AUC, area under the curve; BED, binge eating disorder; BMI, body mass index; ChEAT, Children's Eating Attitudes Test; ChEDE‐Q, Children's Eating Disorder Examination Questionnaire; EAT, Eating Attitudes Test; ED, eating disorder; DEBQ, Dutch Eating Behaviors Questionnaire; EDI, Eating Disorder Inventory; QEWP, Questionnaire for Eating and Weight Patterns; SCOFF, Sick Control One Fat Food.

^a^ Calculated from subscale data.

### 3.1 Measuring change as a continuous variable

When considering change scores (a decrease in scores representing reduced disordered eating to a more normative range, or an increase in scores indicating worsening eating pathology), it is important to understand clinical relevance as well as statistical significance. However, there is no universal definition of a clinically significant change in ED scores, and there is a paucity of literature to provide guidance. One study defined a clinically significant change on the EDE‐Q as 0.45 points in people with anorexia nervosa (Byrne et al., 2017), but this has not been widely applied. Additionally, change in ED scores may not predict negative outcomes in a linear manner. A change from very low to low/medium scores may be less concerning, while a change from medium to high scores may be problematic. More complex nonlinear approaches may be warranted.

The Reliable Change Index (RCI) provides a measure of statistical and clinical significance of an outcome (Jacobson & Truax, 1991). This approach demonstrates how much, and in what direction an individual has changed, and whether that change is reliable and clinically significant (Zahra & Hedge, 2010). Normative data are sparse for people with higher weight, and reliability data are not available for all assessments, making the RCI an important area for future development.

### 3.2 Measuring change as a categorical variable

Applying cut‐points to assessment tools provide guidance on how these can be applied in clinical practice to identify people who may meet diagnostic criteria for an ED, requiring further investigation with a clinical interview. Cut‐points for measuring ED risk in people with higher weight, where available, differ from lower weight samples. For example, Rø et al. (2015) found an optimal cut‐point of 3.15 on the EDE‐Q for people with BMI 25–30 kg/m^2^ and 3.26 for BMI ≥30 kg/m^2^, compared to 2.51 for BMI 18.5–24.9 kg/m^2^, based on a sample of female controls and patients undergoing ED treatment (Table 1). The higher cut‐points appear to be driven by higher weight and shape concerns in people with higher weight (Rø et al., 2012). Comparable data, based on weight status, are not available for adolescents.

When measuring change as a categorical variable, striking a balance between sensitivity and specificity is challenging. Using a lower cut‐point may result in large numbers of people being identified as “high risk” who are not “true positives,” requiring additional resources for assessment via a clinical interview or potential exclusion from obesity treatment. However, this approach does ensure individuals at risk of EDs are identified.

From a statistical point of view, using categorical variables has limitations. First, dichotomizing data means information is lost, so statistical power to detect the relationship between an intervention and patient outcomes is reduced (Altman & Royston, 2006). Second, examining ED risk as a categorical variable may result in underestimating the extent of variation in scores between intervention groups. For example, individuals close to but on opposite sides of a cut‐point are characterized as being very different rather than very similar (Altman & Royston, 2006). Cut‐points may be practical in a clinical setting to identify patients requiring a more detailed clinical assessment, but their utility in a research context has statistical limitations. A multimodal approach, incorporating change as a continuous variable and cut‐points may be beneficial.

## 4 FUTURE DIRECTIONS

**FIGURE 1:**
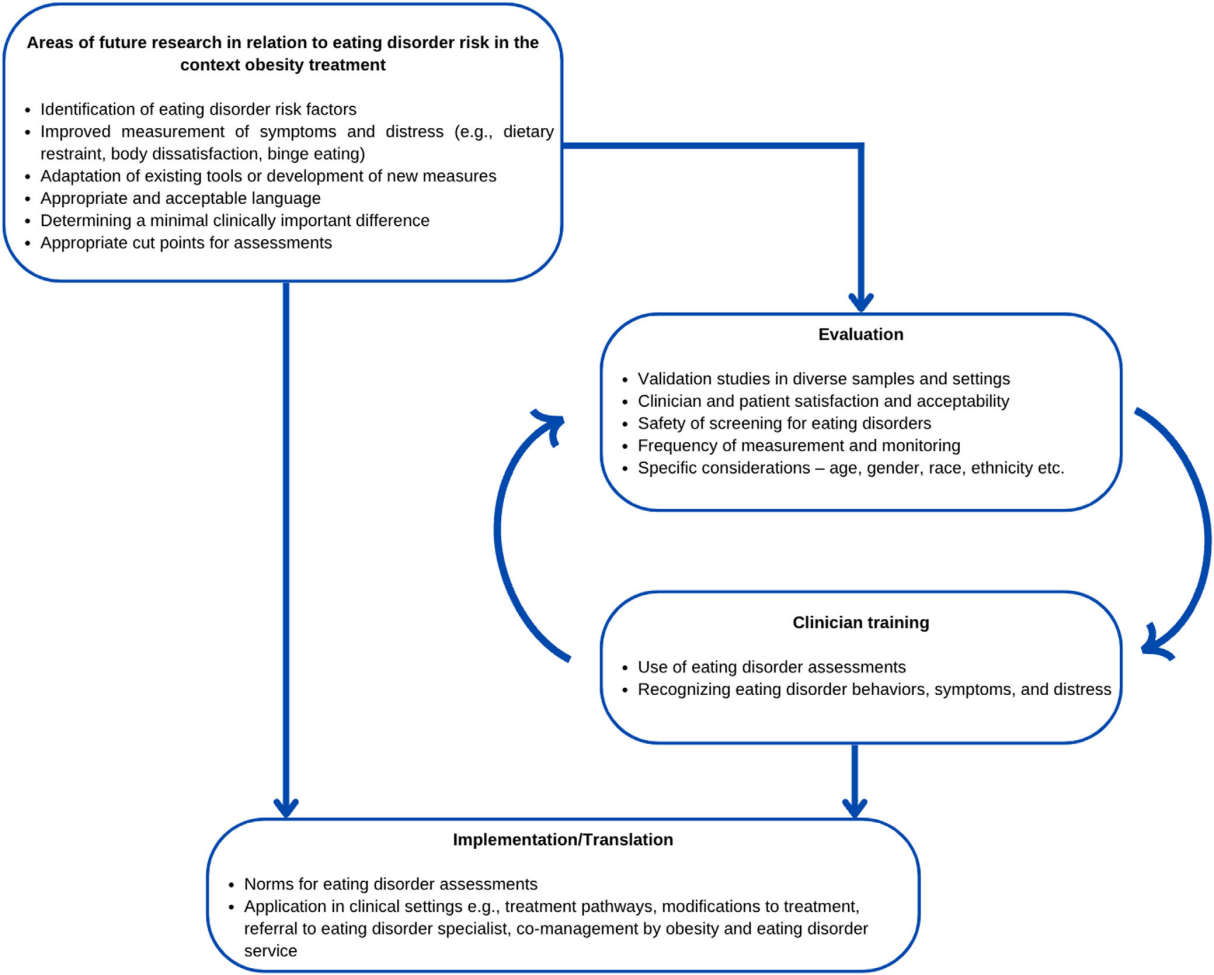
Proposed areas for future research for the assessment and monitoring of eating disorder risk in people with higher weight seeking obesity treatment.

Development of clinical and research protocols for assessment and monitoring of EDs and disordered eating in people with higher weight, across obesity treatment settings, is warranted. Figure 1 proposes a pathway for research and implementation. Research is required to understand how “core” ED behaviors and psychopathology (dietary restraint, body dissatisfaction, binge eating) may differ in presentation among people with higher weight and in an obesity treatment context, and whether there are other risk factors unique to this population or setting. It is important to understand how the onset of disordered eating and the development of EDs differ in an obesity treatment setting compared to what we know of ED development in the community. For a new or adapted assessment, research should explore: (1) Validity in a diverse sample across the weight spectrum including very high weight and across varying severity of comorbidities and different age groups, genders, race and ethnicities, sexual orientations, settings (community, treatment), food environments (with consideration of food insecurity), as well as issues related to intersectionality; (2) Clinician and patient satisfaction and acceptability; (3) Appropriate language with input from people with lived experience of EDs and higher weight; (4) Minimal clinically important difference in ED risk and/or appropriate cut‐point; (5) How to assess change over time to better understand the effects of obesity treatment interventions; and (6) Safety of screening and assessment for EDs in people with higher weight and whether assessment has any detrimental effects, e.g., obesity treatment may not be recommended due to ED concerns and this may be associated with increased metabolic risk. Implementation of assessments across clinical practice and research should be informed by clinical and community norms, and guidance on administration (e.g., frequency of measurements) and a decision tree on how and when to manage ED symptoms and behaviors. These considerations are likely to require tailoring for different population subgroups. Adaptations across different treatment settings, treatment modalities (behavioral weight management, prescriptive dietary approaches, pharmacotherapy, and bariatric surgery) and models of care within different health systems will be required. In particular, primary care settings warrant attention, as weight loss may be recommended with minimal support. ED risk profiles may vary based on the individual and ED risk should be considered alongside indices of physical and mental health.

Implementation of ED assessments specific to an obesity treatment setting could improve early identification and management of EDs in people with higher weight. Additional psychological support within obesity services is needed to assess ED risk using a clinical interview in those identified via self‐report assessments. Linkages with an existing ED service may facilitate these clinical assessments where psychological support is not available within an obesity service. This would improve accuracy of identification, facilitate early intervention to improve treatment outcomes and address biases commonly experienced by people with EDs and higher weight who do not fit cultural stereotypes of EDs. Additionally, establishing clear referral pathways for treatment for people with EDs and higher weight will clarify when specialist ED input is indicated, and when obesity treatment interventions may be beneficial. Accessible treatment options for people with an ED and other health‐related comorbidities are not available in many jurisdictions. For such individuals, specialized multidisciplinary care should be available.

Appropriate assessment, identification, and monitoring of EDs and disordered eating attitudes and behaviors in people with higher weight is important. At present, there is insufficient evidence to guide research and practice. Addressing the identified research gaps will facilitate improved assessment in obesity treatment settings, early identification of EDs, and ultimately improved patient care. This will require greater collaboration between the ED and obesity fields to allow a coordinated approach to research and service delivery.

## AUTHOR CONTRIBUTIONS

**Caitlin M. McMaster:** Conceptualization; investigation; writing – original draft; writing – review and editing. **Susan J. Paxton:** Conceptualization; writing – review and editing. **Sarah Maguire:** Conceptualization; writing – review and editing. **Andrew J. Hill:** Conceptualization; writing – review and editing. **Caroline Braet:** Conceptualization; writing – review and editing. **Anna L. Seidler:** Writing – review and editing. **Dasha Nicholls:** Writing – review and editing. **Sarah P. Garnett:** Writing – review and editing. **Amy L. Ahern:** Writing – review and editing. **Denise E. Wilfley:** Writing – review and editing. **Natalie B. Lister:** Writing – review and editing. **Hiba Jebeile:** Conceptualization; investigation; writing – original draft; writing – review and editing.

## FUNDING STATEMENT

Caitlin M. McMaster is supported by the Australian National Health and Medical Research Council (NHMRC) Ideas Grant (#2002310). Natalie B. Lister is a recipient of a National Health and Medical Research Council Peter Doherty Early Career Fellowship (#1145748). Amy L. Ahern is funded by an NHMRC Emerging Leadership Investigator Grant (#2009432). Dasha Nicholls is supported by the National Institute for Health Research (NIHR) under the Applied Health Research (ARC) programme for Northwest London. The views expressed in this publication are those of the author(s) and not necessarily those of the National Health Service, the NIHR or the Department of Health in England. Amy L. Ahern is supported by the Medical Research Council (MRC) (Grant MC_UU_00006/6). Denise E. Wilfley is supported by the Scott Rudolph University Endowed Professorship at Washington University in St. Louis School of Medicine. Hiba Jebeile is supported by the Sydney Medical School Foundation (University of Sydney) and NHMRC Leadership Investigator Grant (#2009035) awarded to Professor Louise A. Baur.

## CONFLICT OF INTEREST

Amy L. Ahern is Principal Investigator on two publicly funded trials where the intervention is provided by WW (formerly Weight Watchers) at no cost and is a member of the WW Scientific Advisory Board. Andrew J. Hill reports receiving payment for advice given to Slimming World (UK).

## Data Availability

Not available.
